# Acceleration of Diagnosis and Treatment Initiation Using the Elecsys® HCV Duo Assay

**DOI:** 10.7759/cureus.87145

**Published:** 2025-07-01

**Authors:** Tadashi Namisaki, Kiyoko Okuno, Mariko Murai, Yuichiro Eguchi, Takemi Akahane, Hitoshi Yoshiji

**Affiliations:** 1 Department of Gastroenterology and Hepatology, Nara Medical University, Kashihara, JPN; 2 Liver Disease Consultation Center, Nara Medical University, Kashihara, JPN; 3 Department of Gastroenterology, Loco Medical Group, Eguchi Hospital, Kanada, JPN; 4 Department of Gastroenterology, Nara Medical University, Kashihara, JPN

**Keywords:** direct-acting antiviral treatment, hcv antibody, hcv duo immunoassay, hcv rna, simultaneous measurements

## Abstract

This study determined the number of patients with positive hepatitis C virus (HCV) RNA results who were eligible to receive direct-acting antiviral (DAA) therapy at Nara Medical University and identified effective screening methods in this population. Between June 2021 and May 2024, the RNA detection status of HCV antibody (Ab)-positive cases, including RNA detection rates (RNA detection number/antibody-positive number) and positive rates (RNA-positive number/RNA detection number), was evaluated. Among the 866 patients who tested positive for anti-HCV antibodies, HCV RNA testing was performed in 29.2% (n = 253), comprising 57 patients from the Department of Gastroenterology and 196 from other departments. Of these 253 patients, 26 (45.6%) from the Department of Gastroenterology and 16 (8.2%) from other departments tested positive for HCV RNA. RNA positivity was observed in 16.6% of cases (42/253). The remaining 70.8% (n = 613) of antibody-positive patients did not undergo HCV RNA testing. This group included 41 patients from the Department of Gastroenterology and 572 from other departments. Based on the observed HCV RNA positivity rates within each department, it is estimated that among these 613 untested patients, approximately 66 may be HCV RNA-positive, 19 (45.6%) from the Department of Gastroenterology and 47 (8.2%) from other departments. Overall, 66 patients will potentially remain HCV RNA-positive. The simultaneous evaluation of HCV antibodies and HCV RNA can efficiently identify patients with chronic HCV infection who are eligible for DAA therapy.

## Introduction

Chronic hepatitis C virus (HCV) infection is a leading cause of liver cirrhosis, liver failure, and hepatocellular carcinoma (HCC) [1]. The introduction of direct-acting antiviral (DAA) agents has significantly improved treatment outcomes, resulting in high rates of sustained virological response (SVR) in patients with HCV-related chronic liver disease [2].

In Japan, approximately 300,000-600,000 individuals with HCV are treatment-naïve [3]. Although the World Health Organization (WHO) aims to eliminate HCV by 2030, Japan is among the high-income countries projected to meet this target [4]. However, a serious concern is that some individuals who test positive for HCV antibodies (Ab) do not undergo confirmatory HCV RNA testing [5]. Consequently, they are not diagnosed as HCV carriers and miss the opportunity to receive DAA therapy [6]. Nara Prefecture has one of the lowest hepatitis virus testing uptake rates in Japan, indicating that a substantial number of individuals may be unaware of their infection. Therefore, identifying patients eligible for treatment among those who test positive for HCV antibodies is important. Although automated alerts were incorporated into electronic medical records in Nara Medical University in 2016 and checkups of patients with positive HCV antibodies and hepatitis B surface antigen (HBs Ag), as well as monthly pickups by hepatitis medical coordinators, were initiated in 2021, the effectiveness of these efforts remains limited. Thus, effective screening for HCV is essential.

This study aimed to determine the number of patients with positive HCV RNA tests who are eligible to receive DAA therapy at Nara Medical University and to identify effective methods for screening these patients. This study investigated the detection situation of HCV patients in hospitals in specific regions of Japan. By investigating information such as clinical implementation, patient detection rate, and sources, it evaluated patients with clinical benefits and potential infected populations, providing certain data for the early detection and early intervention of this disease.

## Materials and methods

**Figure 1:**
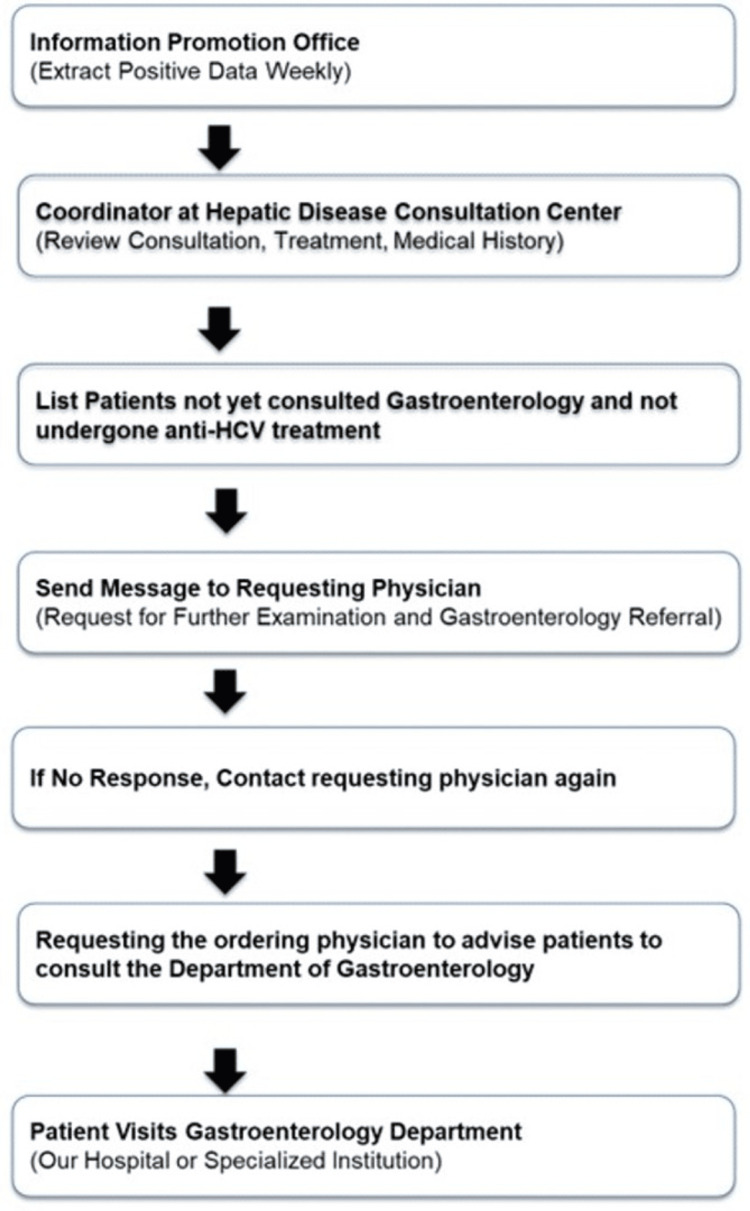
Flow of the In-Hospital Hepatitis Virus Screening System HCV: hepatitis C virus

This is a retrospective cohort study that included 866 patients who tested positive for HCV antibodies between June 2021 and May 2024. Nara Medical University started hepatitis medical coordinator activities in 2015. A total of 866 outpatients who visited Nara Medical University were screened for HCV infection using an anti-HCV antibody (Ab) test. These patients were seen across various departments, including Gastroenterology (n = 440), Ophthalmology (n = 53), Cardiovascular Medicine (n = 39), Digestive Surgery (n = 37), Plastic Surgery (n = 35), Urology (n = 40), Psychiatry (n = 35), Dentistry (n = 25), Oral Surgery (n = 30), Respiratory Medicine (n = 32), Hematology (n = 20), Reconstructive Surgery (n = 30), Obstetrics and Gynecology (n = 30), and Neurosurgery (n = 20). Using the alert function of the electronic medical record, a message was sent to physicians to order tests to confirm chronic hepatitis B virus and HCV infections and to recommend patients with HCV to consult hepatologists and gastroenterologists. In 2021, Nara Medical University implemented the In-Hospital Hepatitis Virus Screening System flow to enhance the detection and management of hepatitis infections in response to the historically low screening rates in Nara Prefecture (Figure 1). This initiative aimed to improve the early diagnosis and treatment outcomes of patients with hepatitis B and C, which contributes to the development of liver disease such as HCC.

HCV antibody and RNA testing

In this study, screening for hepatitis C virus (HCV) infection was performed using the Elecsys® HCV Duo assay (Roche Diagnostics, Basel, Switzerland), an electrochemiluminescence immunoassay (ECLIA) capable of simultaneously detecting HCV antibodies and HCV core antigen. The assay was conducted on the fully automated Cobas® e801 analyzer (Roche Diagnostics, Basel, Switzerland). This dual detection method facilitates the early diagnosis of HCV infection, particularly in cases of recent exposure or immunocompromised patients.

HCV RNA testing was carried out using the Cobas® HCV assay, a quantitative real-time polymerase chain reaction (PCR)-based method implemented on the Cobas® 6800/8800 system. The assay has a lower limit of quantification (LLOQ) of 15 IU/mL and is characterized by high sensitivity and specificity. For patients with detectable HCV RNA, quantitative results were reported in international units per milliliter (IU/mL). The survey items included age, gender, and the presence or absence of cirrhosis*.*

The protocol for this study was approved by the Ethics Committee of Nara Medical University (approval number: Nara 468) and conforms to the provisions of the 1964 Declaration of Helsinki and its later revisions. An opt-out procedure was employed to obtain consent.

Effectiveness of the new In-Hospital Hepatitis Virus Patient Identification System

The In-Hospital Hepatitis Virus Patient Identification System flow is shown in Figure 1. Once a week, we extracted data on patients with positive hepatitis virus test results from the information promotion office. Consultation records, treatments, and medical history of each patient were obtained from the Liver Disease Consultation Center coordinator and thoroughly reviewed. Through consultation with a dedicated hepatologist, the system identified individuals who test positive for HCV antibodies, have not been seen at the Department of Gastroenterology, and have not received anti-HCV treatment. Notifications were sent via the electronic medical record system to physicians in the Departments of Gastroenterology, Ophthalmology, Cardiovascular Medicine, Digestive Surgery, Plastic Surgery, Urology, Psychiatry, Dentistry, Oral Surgery, Respiratory Medicine, Hematology, Reconstructive Surgery, Obstetrics and Gynecology, and Neurosurgery. The doctors recommended further examinations and encouraged referral to the Department of Gastroenterology in collaboration with the hepatitis coordinator. If the response from the ordering physician was unclear, the coordinator contacted the physician for confirmation. The ordering physician then instructed the patient to seek consultation with the Department of Gastroenterology at Nara Medical University Hospital.

Statistical analyses

All statistical analyses were conducted using EZR version 1.68 (Saitama Medical Center, Jichi Medical University, Shimotsuke, Japan) [7]. A one-way analysis of variance (ANOVA) was performed to assess relationships among the groups.A two-sided p-value of <0.05 was considered statistically significant.

## Results

Frequency of referral requests issued to physicians who ordered HCV antibody tests

**Figure 2:**
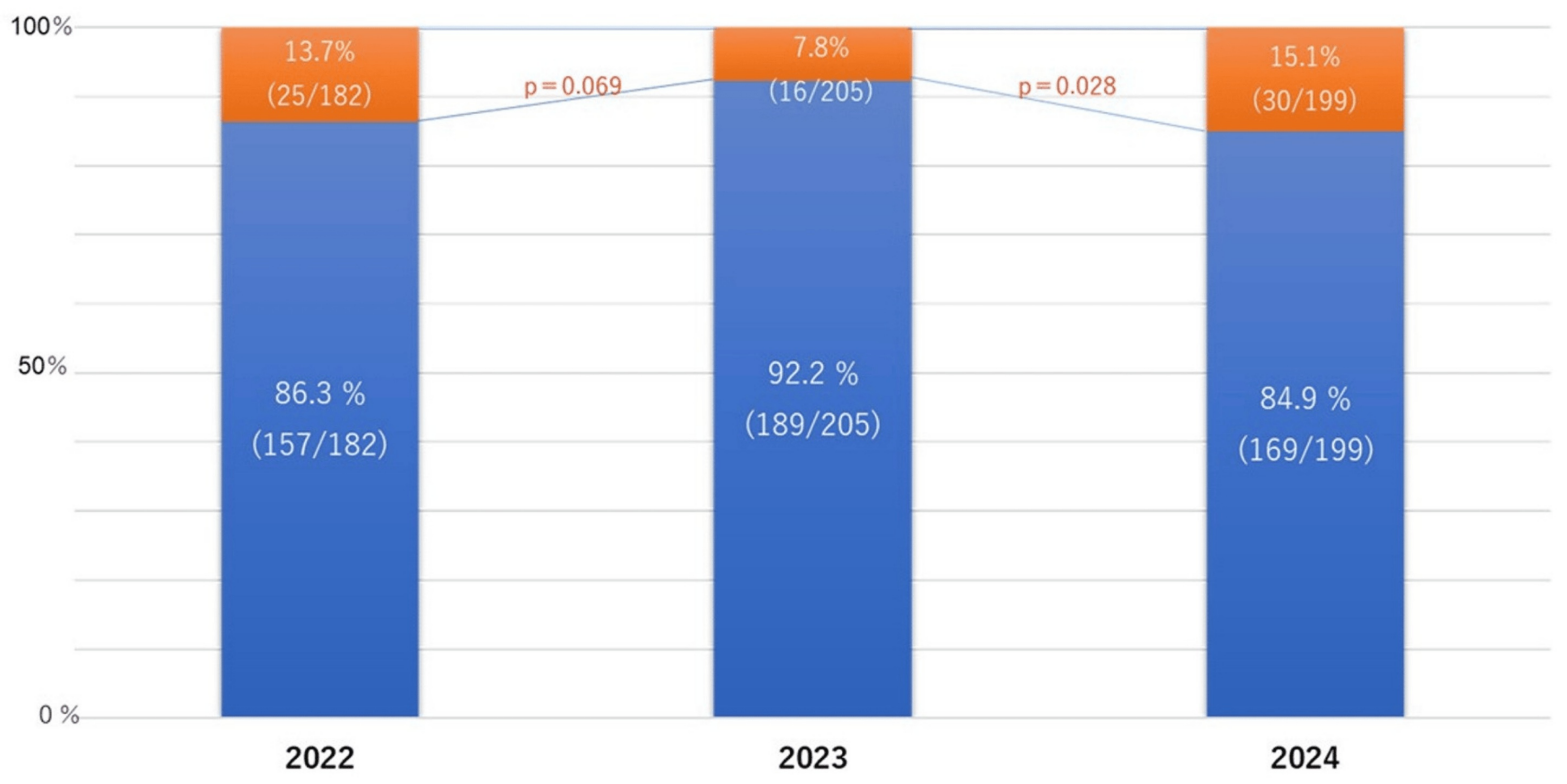
The rates of contacting the physician who ordered the HCV antibody tests between 2022 and 2024 The population includes all patients within the hospital who were tested and found to be positive for HCV antibody. The orange bars represent the number of patients who tested positive for HCV antibody but had no documentation of HCV-related treatment in their medical records. These cases were identified by the Liver Disease Center and referred to other departments for subsequent referral to the Department of Gastroenterology. The blue bar indicates the proportion of cases in which treatment was initiated following physician intervention HCV: hepatitis C virus

Although the consultation rate declined from 13.7% in 2022 to 7.8% in 2023, it subsequently increased significantly to 15.1% in 2024 (Figure 2).

Diagnosing HCV infection

Among the 866 patients who underwent anti-HCV testing and were antibody-positive, HCV RNA testing was performed in 253 (29.2%) patients, 57 from the Department of Gastroenterology and 196 from other departments (Table 1). These 253 patients included those directly managed by physicians in the Department of Gastroenterology, as well as patients whose attending physicians were advised to consult Gastroenterology for further evaluation.

**Table 1:** Patients with a positive anti-HCV test HCV: hepatitis C virus

	Total cases	Gastroenterology	Others
HCV antibody-positive	100% (866)	98	768
HCV RNA measured	29.2% (253)	57	196
HCV RNA not measured	70.8% (613)	41	572

**Figure 3:**
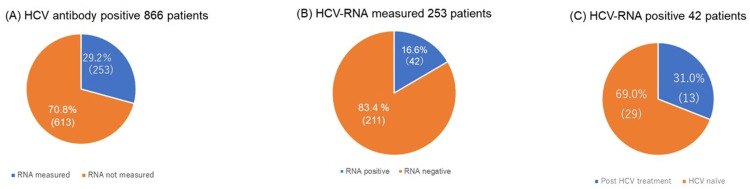
Diagnosing HCV infection (A) Examination rates of HCV RNA tests, (B) positivity of HCV RNA tests, and (C) DAA treatment rates. The blue area represents patients whose HCV RNA was measured, while the orange area represents those whose HCV RNA was not measured HCV, hepatitis C virus; DAA, direct-acting antiviral

Of the 253 patients with a positive anti-HCV test who underwent HCV RNA testing, 42 (16.6%) were confirmed to be HCV RNA-positive. Among these, 26 (45.6%) were from the Department of Gastroenterology, and 16 (8.2%) were from other departments (Table 2 and Figure 3).

**Table 2:** Patients with a positive HCV RNA test HCV: hepatitis C virus

	Total cases	Gastroenterology	Others
HCV RNA-positive rate	16.6% (42/253)	45.6% (26/57)	8.2% (16/196)
Potential RNA-positive rate	10.7% (66/613)	45.6% (19/41)	8.2% (47/572)

Of the 42 patients who were HCV RNA-positive, 29 (69.0%) were treatment-naïve; 15 (51.7%) and 14 (48.3%) were from the Department of Gastroenterology and other departments, respectively. Meanwhile, sustained virological response was achieved in 13 patients (31.0%), indicating successful HCV eradication.

Among the 42 patients who tested positive for HCV RNA, 31.0% (n = 13) received an eight-week course of HCV DAA therapy (Table 3).

**Table 3:** Patients with a positive HCV RNA test HCV: hepatitis C virus

HCV RNA-positive	Total 42 patients	Gastroenterology	Others
Post-HCV elimination	31% (13)	86.4% (11)	15.6% (2)
HCV-naïve	69% (29)	51.7% (15)	48.3% (14)

Potential HCV infection

Meanwhile, the 613 (70.8%) patients who tested positive for anti-HCV antibodies but did not undergo HCV RNA evaluations included 41 patients from the Department of Gastroenterology and 572 patients from other medical departments (Table 1).

Based on the observed RNA positivity rate of 16.6%, an estimated 66/613 patients, comprising 19 (45.6%) from the Department of Gastroenterology and 47 (8.2%) from other departments, were potentially positive for HCV RNA (Table 2).

## Discussion

In this study, we observed that the rate of identified HCV-positive cases initially declined following the introduction of a new hepatitis screening and identification system. However, the contact rate with the examining physicians at the hospital significantly increased.

Based on the observed HCV RNA positivity rates, approximately 66 patients who may require DAA therapy were overlooked. This may be partially explained by insufficient HCV antibody screening and inadequate coordination regarding anti-HCV-positive cases between the hepatitis care coordinator and physicians in other departments [8]. Strengthening referral pathways and incentivizing greater engagement by primary care physicians on hepatitis C management are critical to ensure that screening efforts translate into effective clinical interventions [9].

In 2016, automated alerts were integrated into the electronic medical record system and almost simultaneously implemented across most clinical departments. In 2021, the standardized “Flowchart for HCV Antibody (HCV Ab) and HBs Antigen (HBs Ag) Positive Patients” was established to guide subsequent clinical actions, resulting in effective multidisciplinary collaboration regarding hepatitis care activities. However, despite the immediate review of patients positive for HCV antibodies and HBs antigen and the monthly follow-up by hepatitis medical coordinators, there was a significant increase in the number of cases wherein positive test results were not adequately communicated to the patients. Therefore, “leader coordinators,” which included members from various professions such as a nurse, a pharmacist, and a physical therapist, were introduced, but their effectiveness was limited. These findings highlight systemic shortcomings that undermine early diagnosis and timely treatment, which are critical steps for achieving the national hepatitis C elimination targets. In Germany, initiatives aimed at enhancing the hepatitis C care continuum represent a significant advancement in improving hepatitis C care and include reflex testing, wherein laboratories automatically conduct HCV RNA PCR testing on anti-HCV-positive samples obtained through the “Check-Up” program [10]. As such, further local structural interventions are needed.

Reflex HCV RNA testing is an automated process in which HCV RNA is measured using the same blood sample immediately following a positive anti-HCV antibody result, eliminating the need for additional test orders or blood draws [11]. This approach, currently recommended by the WHO, has been extensively validated and shown to improve the accuracy and timeliness of HCV diagnosis, promote earlier initiation of treatment, and reduce patient loss to follow-up [12,13]. The prevalence of an SVR is high among patients with positive HCV antibody results. The type of HCV RNA test used to confirm the presence of viremia in anti-HCV-positive patients can be guided by the anti-HCV signal-to-cutoff (S/CO) ratio: a ratio of <10.9 indicates the need for qualitative HCV RNA testing, while a ratio of ≥10.9 indicates the need for quantitative HCV RNA testing. However, this finding suggests that, regardless of the HCV antibody titer, additional testing, such as an HCV RNA test, remains necessary to establish active infection. Therefore, the simultaneous measurement of the HCV antibody and core antigen using the HCV Duo immunoassay offers significant medical, time, and cost advantages by eliminating the need for separate HCV RNA testing. The clinical utility of the HCV core antigen has been reported [14].

The Elecsys® HCV Duo assay enables the simultaneous detection of HCV antibodies (Duo/Ab) and core antigen (Duo/Ag) at a reimbursement rate of 102 points, which is equivalent to that of conventional anti-HCV antibody testing (approximately USD 6.84) [15]. The Elecsys® HCV Duo immunoassay, which was introduced in 2022, simultaneously detects HCV antibodies and the HCV core antigen, with the results being available in approximately 27 minutes. A positive result for the HCV core antigen can already confirm active HCV infection, potentially minimizing the reliance on HCV RNA testing. This diagnostic tool may facilitate earlier medical intervention and increase the number of patients receiving antiviral therapy. Additionally, this assay can identify approximately 80% of patients with active HCV infection even without performing confirmatory HCV RNA testing, thereby streamlining the diagnostic process and reducing the time to treatment initiation [16]. These findings indicate that the Duo assay can substantially reduce the costs associated with HCV RNA testing (insurance score: 412 points) for identifying HCV carriers and that up to 85% of previous follow-up testing may have been unnecessary. However, even when the HCV Duo assay yields a positive antibody result and a negative antigen result, HCV RNA may still be detectable. Therefore, confirmatory HCV RNA testing remains essential. Employing the Duo assay may also significantly reduce the workload for healthcare personnel, including physicians, administrative staff, nurses, and hepatitis care coordinators [17]. As it has a rapid turnaround time of 27 minutes and costs similar to that of standard HCV Ab testing, the Duo assay offers a cost-effective and time-efficient strategy for the early identification of patients with HCV[18]. Through same-day confirmation of active infection, the HCV Duo assay may also potentially reduce diagnostic delays, streamline care pathways, and enhance referral rates to specialized departments [19]. Moreover, integrating the Duo assay into routine screening programs could substantially improve the linkage to care and support national efforts toward HCV elimination [20].

**Figure 4:**
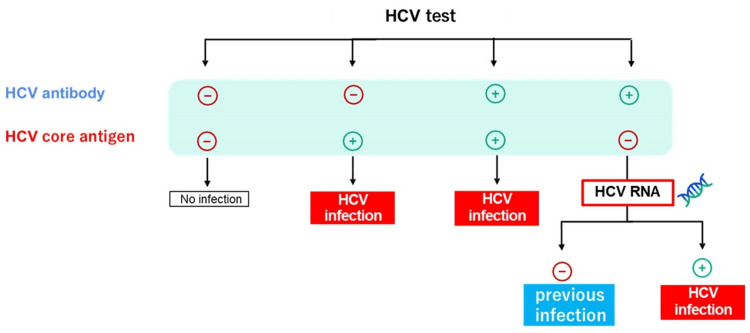
Diagnostic interpretation of hepatitis C virus antibody (HCV Ab) and HCV core antigen (Ag) test results using the Elecsys® HCV Duo assay Patients with confirmed HCV infection, indicated by a positive HCV core antigen or HCV RNA result, should be considered for treatment with direct-acting antiviral (DAA) therapy in accordance with current clinical guidelines

This study has several limitations. First, the study was conducted retrospectively and had a small sample size. Second, the cohort consisted exclusively of Japanese patients and only included HCV genotypes 1 and 2. Third, the estimated number of patients with positive HCV RNA tests was only a theoretical projection and did not correspond to the actual number of individuals. The HCV Duo assay is strongly recommended for hepatitis C virus testing, as it enables the simultaneous detection of HCV core antigen and antibodies, thereby facilitating early and accurate diagnosis (Figure 4). As a result, the implementation of the previously used In-Hospital Hepatitis Virus Patient Identification System is no longer necessary. Patients with confirmed HCV infection, indicated by a positive HCV core antigen or HCV RNA result, should be considered for treatment with direct-acting antiviral (DAA) therapy in accordance with current clinical guidelines.

## Conclusions

In conclusion, among 613 cases that tested positive for the HCV antibody, 66 patients would remain HCV RNA-positive based on the RNA positivity rates observed at the respective facilities. The simultaneous measurement of the HCV antibody and core antigen by the HCV Duo immunoassay offers significant medical, temporal, and economic advantages by eliminating the need for separate HCV RNA testing. The Duo assay may serve as a useful screening tool to support efforts toward HCV elimination, aiding in the diagnosis of chronic HCV infection and facilitating the timely initiation of DAA therapy.
